# Association of Power Outage With Mortality and Hospitalizations Among Florida Nursing Home Residents After Hurricane Irma

**DOI:** 10.1001/jamahealthforum.2021.3900

**Published:** 2021-11-24

**Authors:** Julianne Skarha, Lily Gordon, Nazmus Sakib, Joseph June, Dylan J. Jester, Lindsay J. Peterson, Ross Andel, David M. Dosa

**Affiliations:** 1 School of Public Health, Brown University, Providence, Rhode Island; 2 Department of Industrial and Management Systems Engineering, University of South Florida, Tampa; 3 Florida Policy Exchange Center on Aging, School of Aging Studies, University of South Florida, Tampa; 4 Department of Psychiatry, University of California San Diego, La Jolla; 5 Sam and Rose Stein Institute for Research on Aging, University of California San Diego, La Jolla; 6 Warren Alpert Medical School, Brown University, Providence, Rhode Island; 7 Department of Primary Care, Providence VAMC, Providence, Rhode Island

## Abstract

**Importance:**

Exposure to hurricanes is associated with increased mortality and morbidity in nursing home (NH) residents, but the factors contributing to these outcomes are less understood. One hypothesized pathway could be power outages from hurricanes that expose NH residents to excess ambient heat.

**Objective:**

To determine the association of power loss from Hurricane Irma with hospitalization and mortality in NH residents in Florida.

**Design, Setting, and Participants:**

This retrospective cohort study of NH residents residing in Florida when Hurricane Irma landed on September 10, 2017, assessed mortality at 7 and 30 days after the storm and hospitalization at 30 days after the storm. The analysis was conducted from May 2, 2021, to June 28, 2021. All NH residents residing in Florida at landfall were eligible (N = 67 273). We excluded those younger than 65 years, missing power status information, or who were evacuated (13 178 [19.6%]).

**Exposure:**

We used state-administered surveys to determine NH power outage status. Exposed residents experienced a power outage poststorm, whereas unexposed residents did not experience a power outage poststorm.

**Main Outcomes and Measures:**

We used Medicare claims to assess mortality and hospitalization after Hurricane Irma landfall using generalized linear models with robust standard errors.

**Results:**

In the aftermath of Hurricane Irma, 27 892 residents (18 510 women [66.4%]; 3906 [14.0%] Black, 1651 [5.9%] Hispanic, and 21 756 [78.0%] White individuals) in 299 NHs were exposed to power loss and 26 203 residents (17 620 women [67.2%]; 4175 [15.9%] Black, 1030 [3.9%] Hispanic, and 20 477 [78.1%] White individuals) in 292 NHs were unexposed. Nursing homes that lost power were similar in size, quality star rating, and type of ownership compared with NHs that did not lose power. Power loss was associated with an increased adjusted odds of mortality among all residents within 7 days (odds ratio [OR],1.25; 95% CI,1.05-1.48) and 30 days (OR, 1.12; 95% CI,1.02-1.23) poststorm and hospitalization within 30 days, although only among residents aged 65 to 74 years (OR, 1.16; 95% CI, 1.03-1.33).

**Conclusions and Relevance:**

In this cohort study, power loss was associated with higher odds of mortality in all affected NH residents and hospitalization in some residents. The benefits and costs of policies that require NHs to have emergency alternate power sources should be assessed.

## Key Points

**Question:**

What is the association of a power outage after a hurricane with hospitalization and mortality among nursing home residents?

**Findings:**

In this retrospective cohort study of 54 095 nursing home residents in Florida, more than half of residents experienced power loss when Hurricane Irma landed in 2017. Power loss was associated with an increased odds of mortality within 7 days and 30 days after the storm.

**Meaning:**

The study results suggest that further research should assess the benefits and costs of policies that require nursing homes to have emergency alternate power sources.

## Teaser

This cohort study examines the association of power loss from Hurricane Irma with hospitalization and mortality in nursing home residents in Florida.

## Introduction

In September 2017, Rehabilitation Center at Hollywood Hills, a nursing home (NH) near Miami, Florida, received national attention when 12 of its residents died in the aftermath of Hurricane Irma.^1^ A medical examiner ruled heat exposure as the primary cause of death in many of the fatalities after the otherwise unaffected facility lost power to its air conditioning units, allowing ambient temperatures in the building to rise to dangerous levels. In response to the incident, the state of Florida expedited a power rule mandating that all long-term care facilities have alternate power sources to run air conditioner units.^2^ Although mandated in Florida, to our knowledge, few other states with similar hurricane risk have adopted such legislation because of facility-borne costs and an absence of data to support the added expense.

Previous studies show that exposure to major hurricanes is associated with increased risk of mortality, hospitalization, and subsequent functional decline among NH residents.^3,4^ Further investigation of these events established that the transfer trauma inherent in evacuation may increase the risk of adverse health effects.^5,6,7^ For example, during Hurricanes Katrina, Rita, Gustav, and Ike, NH residents who experienced an evacuation had a greater risk of mortality compared with those who sheltered in place.^8^ Although sheltering in place might be the preferred approach for many NH administrators, staying put may expose residents to power outages for prolonged periods.^9^

To our knowledge, there is little recent research on the effects of power outages on NH residents in the US. Prolonged exposure to heat has been associated with adverse health outcomes in epidemiological studies of heat waves in community populations. A case-control study by Semenza et al^10^ found that those who were bed bound, unable to care for themselves, or lived alone were significantly more likely to die of heat-related mortality during the severe 1995 heat wave in Chicago, Illinois. However, those with access to air conditioning units were protected. Similar findings happened in Europe during a 2003 heat wave that killed thousands, disproportionally affecting older adults.^11^ Furthermore, research on the August 2003 power blackout in New York, New York, found that power loss was associated with an increase in mortality, particularly among community-dwelling older adults.^12,13^

Nursing home residents are considered a vulnerable population because of their high prevalence of comorbidities and functional impairment. There are more than 1 million NH residents in the US, and about 40% are 85 years or older.^14^ Nearly all NH residents require assistance with activities of daily living, and almost half have a diagnosis of Alzheimer disease or related dementias.^14^ Because of this unique medical vulnerability, as well as the increase in hurricane storm intensity and frequency owing to climate change, we aimed to estimate the associations of power loss with hospitalization and mortality among NH residents in the state of Florida during the aftermath of Hurricane Irma.^15^

## Methods

### Study Population

Hurricane Irma made landfall in Cudjoe Key, Florida, at 9:10 am on September 10, 2017.^16^ Because of the storm’s extensive size of more than 400 miles wide, we assumed that all NH residents in Florida on the date of landfall were affected by the storm, as has been done with previous Hurricane Irma health services research.^4,17^ We used data from the US Centers for Medicare & Medicaid Services (CMS) to obtain information on all NH residents who were residing in Florida on the landfall date. More specifically, we used the Minimum Data Set 3.0 for demographic variables for the residents, such as age and sex, as well as a mortality predictor and a disability measure. The Changes in Health, End-Stage Disease and Symptoms and Signs scale was developed to predict mortality in nursing home settings and is strongly associated with hospitalization.^18^ The Activities of Daily Living (ADL) measure was designed to assess limitations with activities associated with personal care, such as bathing, walking, and eating, and is also associated with hospitalization and mortality.^19^ We also abstracted information on participant race and ethnicity from the Minimum Data Set that uses a combination of self-report and other databases, like US Social Security, to create a race classification variable. We used the standard analytical files for Medicare claims, which allowed us to determine hospitalization or mortality. Because of regulations on how Medicare claims are processed, we could ascertain only whether a NH resident was hospitalized within a 30-day period after landfall. We chose to focus on first hospitalization only and did not include repeated hospitalizations, as this may be indicative of greater health issues that are not associated with power loss. We repeated this 30-day outcome with mortality and also included a 7-day mortality outcome because of previous literature showing the length of the effects of power loss to be around 1 week.^20^

There was a total of 67 273 NH residents in Florida on September 10, 2017. We excluded residents who were younger than 65 years 6271 [9.3%]), as they did not qualify for Medicare. Institutional review boards at Brown University and the University of South Florida approved this study. Because this was an analysis of secondary data, the institutional review boards waived informed consent. We followed the Strengthening the Reporting of Observational Studies in Epidemiology (STROBE) reporting guidelines checklist for cohort studies.

### Power Outage Status

Data concerning whether NHs lost power were available from 2 sources. Florida state law required NHs to give a daily report of key data (eg, evacuation, power outage status) before and after the hurricane through an online system called FLHealthSTAT. While all NHs complied, there were inconsistencies in daily reporting and confusion on how to report during the storm, and some NH operators were unable to complete the report because of power loss. The Florida Agency for Health Care Administration gathered additional evacuation and power loss data using the Hurricane Irma Facility Impact (HIFI) survey sent as an official request, although completion was voluntary. The data were collected 6 weeks after the storm when NH operators had the time to review their records, which we believe improved the accuracy in reporting power loss. Thus, for the 302 facilities that completed the HIFI survey, we used the HIFI data to determine whether NHs lost power; for the remaining 376 facilities, we used the FLHealthSTAT data. To check the comparability of these data, we looked at NHs that completed the FLHealthSTAT and HIFI survey and found that 12 NHs (4.0%) had contradictory power information (eg, one reported power loss while the other reported power maintained). For these 12, we treated the NH as lost power regardless of the source that indicated power loss.

We excluded 2 facilities that were missing power information (n = 176 residents) and 85 facilities that evacuated their residents either before or after landfall, as we did not know the power loss status at the relocation site (n = 6731 residents). Of the 206 NH administrators who completed the HIFI survey and reported date of power loss, 125 (61%) lost power on September 10, 2017; 58 (28%) lost power on September 11, 2017; and 23 (11%) lost power after September 11, 2017. Using this information, we created 2 groups of residents: residents living in 299 NHs that reported initial storm-related power loss for any length of time between September 10 and September 15, 2017 (n = 27 892 residents), and residents living in 292 NHs that did not report any power loss during this period (n = 26 203 residents).

### Covariates

We used a directed acyclic graph (eFigure 1 in the Supplement) to identify variables we expected to be associated with NH facility power loss and resident hospitalization and mortality.^4^ Specifically, we determined NH owner status (for profit, not for profit, government run), number of beds within the NH, overall NH star rating (range, 1-5 [higher indicating better quality]), and rural county indicator to be potential confounders. We obtained the overall NH quality star rating from the NH Compare Five Star Quality Rating data reported by CMS. The CMS also provided the Certification And Survey Provider Enhanced Reports data system that we used to obtain the remaining variables. We adjusted for these factors in our analyses.

### Statistical Analyses

We calculated descriptive statistics of the NH residents by power loss status and estimated *P* values using a 2-sample test for equality of proportions (*z* test) with continuity correction for count values and, given unequal variances, the Welch 2-sample *t* test for mean values. We also used the same descriptive statistics to assess differences in facility-level characteristics by power loss status and calculated the respective *P* values.

To estimate the unadjusted and covariate-adjusted association between power loss status and hospitalization and mortality while accounting for correlations within NH, we used generalized linear models (GLMs) with a binomial family, logit link, and robust standard errors that were adjusted for clustering within NHs by including NH identifiers. We adjusted for the potential confounders described in the covariate section. To determine statistical significance for all analyses, we used a type I error rate of .05. We conducted the analysis from May 2, 2021, to June 28, 2021. All analyses were performed in R, version 4.0.1 (R Foundation).

### Effect Size Modification

Previous literature has established differences in long-stay and short-stay residents. Long-stay residents (in the NH ≥90 days) tend to be older, have higher baseline mortality and increased comorbidities, including dementia, and are less likely to be hospitalized compared with short-stay residents (in the NH <90 days).^21^ Thus, using the same GLMs, we examined possible effect size modification by resident stay status. We also estimated effect size modification by age group because of substantial research on the association between age, heat, and mortality and hospitalization. We calculated total counts of first hospitalizations within 30 days and the number of deaths within 7 days and 30 days by stay status and age group.

### Sensitivity Analysis

To determine the robustness of the GLM cluster model, we ran a sensitivity analysis using a mixed effects model. We used a logistic mixed effects model with a specification of a random effect for NH ID (to adjust for clustering) and estimated unadjusted and covariate-adjusted odds ratios (ORs) of first hospitalization and mortality.

## Results

In the study population (N = 54 095), most residents were women (36 130 [67%]), White 42 233 [78%]), and long-stay residents 37 902 [70%]). Compared with residents who did not experience power loss, residents in NHs with reported power loss were less likely to be Black (14.0% vs 15.9%; *P* < .001) and more likely to be older than 85 years (46.7% vs 43.5%; *P* < .001) (Table 1). For the NHs that were used in this study (N = 591), most were for profit (437 [74%]) with a mean (SD) NH Compare Star rating of 3.77 (1.26) and a mean (SD) bed count of 124 (49); 36 NHs (6.1%) were located in a rural county. No appreciable differences were noted between NHs that lost power and those that maintained power (Table 2).

**Table 1.** Baseline Demographic and Health Characteristics of Nursing Home Residents in Florida by Power Loss Status After Hurricane Irma^a^

Characteristic	Reported power loss, No. (%)		*P* value
	Yes (n = 27 892)	No (n = 26 203)	
Sex			
Women	18 510 (66.4)	17 620 (67.2)	.03
Men	9382 (33.6)	8583 (32.8)	.03
Age, y			
65-74	5747 (20.6)	6093 (23.3)	<.001
75-84	9107 (32.7)	8707 (33.2)	.16
≥85	13 038 (46.7)	11 403 (43.5)	<.001
Race			
Black	3906 (14.0)	4175 (15.9)	<.001
Hispanic	1651 (5.9)	1030 (3.9)	<.001
White	21 756 (78.0)	20 477 (78.1)	.69
Other^b^	442 (1.6)	410 (1.6)	.88
Missing	137 (0.5)	111 (0.4)	NA
Resident acuity measures, mean (SD)^c^			
CHESS	0.762 (0.94)	0.712 (0.92)	<.001
Missing	4995 (17.9)	4779 (18.2)	
ADL	18.1 (5.69)	17.5 (5.88)	<.001
Missing	4064 (14.6)	3867 (14.8)	
Stay type			
Long stay	19 598 (70.3)	18 304 (69.9)	.30
Short stay	8294 (29.7)	7899 (30.1)	.30

Abbreviations: ADL, activity of daily living; CHESS, changes in health, end-stage disease, and signs and symptoms.

^a^ To derive *P* values for count values, we used a 2-sample test for equality of proportions with continuity correction. To derive *P* values for mean values, we used the Welch 2-sample *t* test.

^b^ Other race includes Asian/Pacific Islander and Native American.

^c^ CHESS (range, 0-5 [ higher scores indicating worse prognosis]); ADL (range, 0-28 [higher scores indicate more dependent]).

**Table 2.** Characteristics of NHs in Florida by Power Loss Status After Hurricane Irma^a^

Characteristic	Reported power loss, No. (%)		*P* value
	Yes (n = 299)	No (n = 292)	
NH ownership			
For profit	213 (71.2)	222 (76.0)	.21
Not for profit	77.0 (25.8)	68.0 (23.3)	.55
Government run	9 (3.0)	2 (0.7)	.07
NH star rating, mean (SD)	3.84 (1.22)	3.70 (1.30)	.19
NH bed count, mean (range)^b^	125 (20.0-438)	122 (22.0-310)	.34
Rural	18 (6.0)	18 (6.2)	>.99

Abbreviation: NH, nursing home.

^a^ To derive *P* values for count values, we used a 2-sample test for equality of proportions with continuity correction. To derive *P* values for mean values, we used the Welch 2-sample *t* test.

^b^ We reported a minimum and maximum for bed count to better depict the range of facility sizes, as this variable was not normally distributed.

We found that power loss was not associated with hospitalization (OR, 1.05; 95% CI, 0.96-1.14) but was associated with an increased adjusted odds of mortality within 7 days (OR, 1.25; 95% CI, 1.05-1.48) and 30 days (OR, 1.12; 95% CI, 1.02-1.23) after hurricane landfall. These results changed minimally with covariate adjustment (Table 3). This increase in mortality can also been seen in eFigure 2 in the Supplement of the daily mortality rate around Hurricane Irma landfall by power loss status.

**Table 3.** Adjusted and Unadjusted Odds Ratios of First Hospitalization Within 30 Days and Mortality Within 7 and 30 Days Among NH Residents Who Experienced vs Did Not Experience a Power Loss After Hurricane Irma^a^

Characteristic	Odds ratio (95% CI)	
	Unadjusted	Adjusted
First hospitalization within 30 d	1.03 (0.94-1.13)	1.05 (0.96-1.14)
Mortality within 7 d	1.25 (1.05-1.48)	1.25 (1.05-1.48)
Mortality within 30 d	1.10 (1.00-1.21)	1.12 (1.02-1.23)

^a^ We used generalized linear models with a binomial family and robust standard errors that were clustered for nursing homes. In the adjusted models, we adjusted for nursing home owner status (for profit, not for profit, and government run), continuous bed count of nursing home beds, overall nursing home star rating (1-5), and rural county indicator.

### Effect Size Modification

The association between power loss and hospitalization did not change by stay status (short stay OR, 1.00; 95% CI, 0.92-1.10; vs long stay OR, 1.08; 95% CI, 0.96-1.21; interaction *P* = .23) but did change by age category (interaction *P* = .01). Residents aged 65 to 74 years had 1.16 times the adjusted odds of hospitalization (95% CI, 1.03-1.33) of residents aged 65 to 74 years who did not experience power loss (Table 4). We found no difference in power loss and mortality within 7 and 30 days by either stay status or age category.

**Table 4.** Adjusted Odds Ratios of First Hospitalization Within 30 Days and Mortality Within 7 and 30 Days Among NH Residents Who Experienced vs Did Not Experience a Power Loss After Hurricane Irma by Residential Characteristics^a^

Characteristic	Adjusted odds ratio (95% CI)		
	First hospitalization within 30 d	Mortality within 7 d	Mortality within 30 d
Stay type			
Short stay	1.00 (0.92-1.10)	1.23 (0.96-1.57)	1.08 (0.95-1.23)
Long stay	1.08 (0.96-1.21)	1.26 (1.01-1.57)	1.15 (1.01-1.31)
Age group, y			
65-74	1.16 (1.03-1.33)	1.18 (0.75-1.87)	1.21 (0.97-1.52)
75-84	0.95 (0.85-1.07)	1.21 (0.90-1.65)	1.17 (1.00-1.37)
≥85	1.09 (0.96-1.24)	1.25 (1.00-1.56)	1.04 (0.92-1.17)

^a^ We used generalized linear models with a binomial family and robust standard errors that were clustered for nursing home. We adjusted for nursing home owner status (for profit, not for profit, and government run), continuous bed count of nursing home beds, overall nursing home star rating (1-5), and rural county indicator.

### Sensitivity Analysis

The results of the mixed effects model were very similar to the results from the GLM clustered model (eTable in the Supplement). Power loss was not associated with hospitalization (OR, 1.05; 95% CI, 0.96-1.14) but was associated with an increased adjusted odds of mortality within 7 days (OR, 1.24; 95% CI, 1.05-1.47) and 30 days (OR, 1.12; 95% CI, 1.02-1.23) after hurricane landfall. As with the GLM cluster models, these results changed minimally with covariate adjustment.

## Discussion

In this cohort study of Florida NH residents in the aftermath of Hurricane Irma, power loss was associated with an increased odds of mortality within 7 and 30 days, with a roughly 25% increase in mortality at 7 days after landfall and about a 10% increase 30 days after landfall. Neither age nor stay status significantly modified these results. Power loss was also associated with an increased odds of first hospitalization among residents aged 65 to 74 years old.

While there is limited recent literature on the association of power loss with NH populations, our results are consistent with previous epidemiological research in other US populations. Anderson and Bell^12^ similarly used GLMs to investigate the risk of mortality in New York, New York, after the 2-day power outage in August 2003. They found that nonincidental deaths increased by 25% and the risk of mortality remained elevated throughout the month. Dominianni et al^22^ also studied the 2003 blackout in New York, New York, and found that the mortality peaked 1 day after the outage. The different daily risk of mortality after power loss may explain some of the difference that we found in the 7-day and 30-day mortality estimates, with the 7-day association estimate capturing more of the immediate effect of the power outage. Additionally, Dominianni et al^22^ investigated 2 other power outages in New York, New York, and found that hospitalizations increased during and after the outages with relative risks ranging between 1.14 and 2.26. Lin et al^13^ reported an odds ratio of 2.58 for hospitalizations among adults 75 years or older after the 2003 New York, New York, blackout. However, in this study, we found an association with power loss and hospitalization only among residents aged 65 to 74 years. This raises a question of whether facilities knowingly or unknowingly prioritize younger residents for transfer out of the facility during heat emergencies instead of adults 75 years or older who may be at an increased risk to heat exposure and adverse heat-related illness.^23^

To our knowledge, these findings provide some of the first epidemiologic evidence supporting the adoption of policies that require emergency power alternatives in NH settings. After Hurricane Irma, Florida enacted legislation requiring NHs to have emergency power sources and additional fuel capable of providing air conditioning for at least 96 hours.^2^ However, related research suggests that meeting these requirements was challenging for many facilities, including for-profit and chain-affiliated facilities.^24^ The Florida legislature initially estimated the 5-year costs to come into compliance at $121 380 545 for the NH industry.^25^ Still, qualitative interviews among NH administrators compliant with power rule credited generators with enabling them to ensure the safety of their residents amid a power loss.^26^ Since 2016, CMS has similarly required medical institutions participating in Medicare to follow emergency preparedness requirements, including alternative power sources and safe evacuation plans.^27^ However, as recently as September 2021, 12 resident deaths occurred after 7 NHs evacuated to a warehouse during Hurricane Ida.^28^ Further research should focus on the specific barriers NHs are encountering when implementing these mandatory emergency plans and how resources may be redirected to accommodate them.^29^

### Strengths and Limitations

This study has several strengths. We analyzed all NH residents in the state of Florida with the exclusion of 2 facilities where power loss information was not available and 85 facilities where residents were evacuated. This may increase the generalizability of this study to other NH populations that experience hurricanes and shelter in place. Because we assumed that any facility that reported power loss lost their power on the day of landfall, there is the possibility that we assigned exposure before it happened. However, only 12% of NH administrators who completed the HIFI survey reported power loss after September 11, 2017, which suggests that the exposure misclassification is likely minimal. Finally, we controlled for possible confounding variables that minimally affected the results and reported similar results from the sensitivity analyses.

There are several limitations to this research. A key limitation of this research is that we were unable to determine the length of a facility’s power outage. This information was not collected in the FLHealthSTAT survey and was missing for most facilities in the HIFI survey. Theoretically, an NH that reported power loss in the FLHealthSTAT survey may have lost power for a short time. However, of the NHs that did report time length of power loss in the HIFI survey, 63 (30.6%) reported power loss for more than 2 days and 50 (24.3%) for more than 24 hours.

Another limitation is that because of missing data, we could not account for generator usage in NHs. It may be that our inability to account for generator use could underestimate the true association of power loss with NH residents in our sample. Additionally, by excluding residents who were evacuated, we may not be capturing the full association of power loss–related mortality. Nursing homes may have evacuated their residents because of power loss, as was the case with the Rehabilitation Center at Hollywood Hills where 12 people died. However, it is also likely that this exclusion may underestimate the association of power loss with mortality.

Finally, another explanation for the increased mortality rate in NHs that lost power could be the quality of care delivered within these facilities. Prior work suggests that NHs increased their direct-care staff in anticipation of Hurricane Irma,^30^ but the change in direct-care staff was largest among high-quality facilities and smallest among low-quality facilities when using the NH Compare overall quality star rating.^31^ The NH Compare overall quality star rating is also strongly associated with resident mortality and preventable hospitalization.^32,33^ By adjusting for the quality star rating, we likely partially controlled for variation in staffing resources. Nevertheless, this still remains a plausible conclusion.

## Conclusions

The findings from this retrospective cohort study suggest that hurricane-related power loss is associated with increased hospitalization of NH residents aged 65 to 74 years and increased mortality for NH residents 65 years or older. Future research should focus on the benefits and costs of this new policy and barriers NHs encounter when implementing potentially lifesaving emergency energy plans.

## Notes

Supplement. **eFigure 1.** Direct Acyclic Graph (DAG) for the relations between power loss, first hospitalization and mortality, and covariates **eFigure 2. **Daily mortality rate of NH residents that experienced a power loss and daily mortality rate of NH residents that did not experience power loss over a 7-day period **eTable. **Unadjusted and adjusted odds ratio from mixed effects model of first hospitalization within 30 days or mortality within 7 and 30 days among NH residents that experienced a power loss after Hurricane Irma compared to residents that did not experience a power loss
