# Supplementary material for: Association of Power Outage With Mortality and Hospitalizations Among Florida Nursing Home Residents After Hurricane Irma
Source: JAMA Health Forum. 2021 Nov 24;2(11):e213900. doi: 10.1001/jamahealthforum.2021.3900 (PMC8796882; doi:10.1001/jamahealthforum.2021.3900)
Supplement: Supplement. — eFigure 1. Direct Acyclic Graph (DAG) for the relations between power loss, first hospitalization and mortality, and covariates eFigure 2. Daily mortality rate of NH residents that experienced a power loss and daily mortality rate of NH residents that did not experience power loss over a 7-day period eTable. Unadjusted and adjusted odds ratio from mixed effects model of first hospitalization within 30 days or mortality within 7 and 30 days among NH residents that experienced a power loss after Hurricane Irma compared to residents that did not experience a power loss [file jamahealthforum-e213900-s001.pdf]

# Supplemental Online Content

Skarha J, Gordon L, Sakib N, et al. Association of power outage with mortality and hospitalizations among Florida nursing home residents after Hurricane Irma. *JAMA Health Forum*.
2021;2(11):e213900. doi:10.1001/jamahealthforum.2021.3900

**eFigure 1.** Direct Acyclic Graph (DAG) for the relations between power loss, first hospitalization and mortality, and covariates

**eFigure 2.** Daily mortality rate of NH residents that experienced a power loss and daily mortality rate of NH residents that did not experience power loss over a 7-day period

**eTable.** Unadjusted and adjusted odds ratio from mixed effects model of first hospitalization within 30 days or mortality within 7 and 30 days among NH residents that experienced a power loss after Hurricane Irma compared to residents that did not experience a power loss

This supplemental material has been provided by the authors to give readers additional information about their work.

© 2021 Skarha J et al. *JAMA Health Forum.*

**eFigure 1: Direct Acyclic Graph (DAG) for the relations between power loss, first hospitalization and mortality, and covariates**

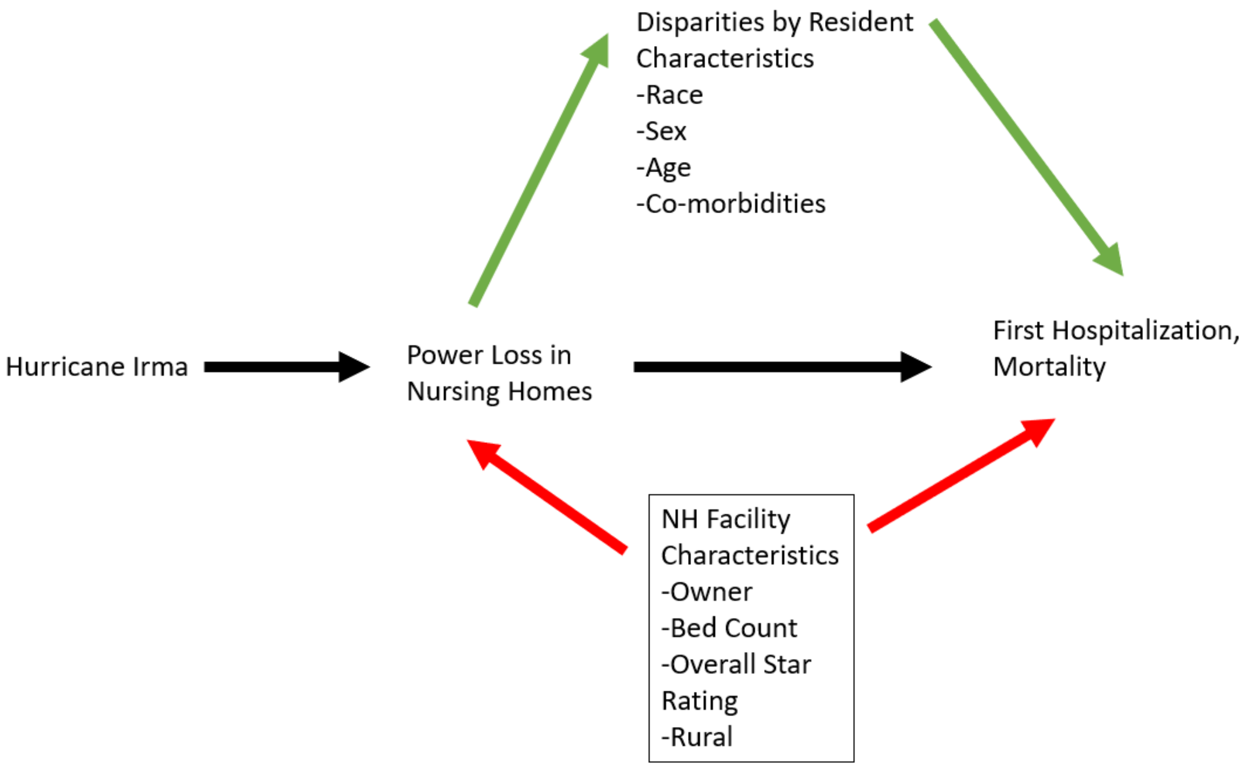


© 2021 Skarha J et al. *JAMA Health Forum.*

**eFigure 2: Daily mortality rate of NH residents comparing those that experienced a power loss and those that did not over a 7-day period**

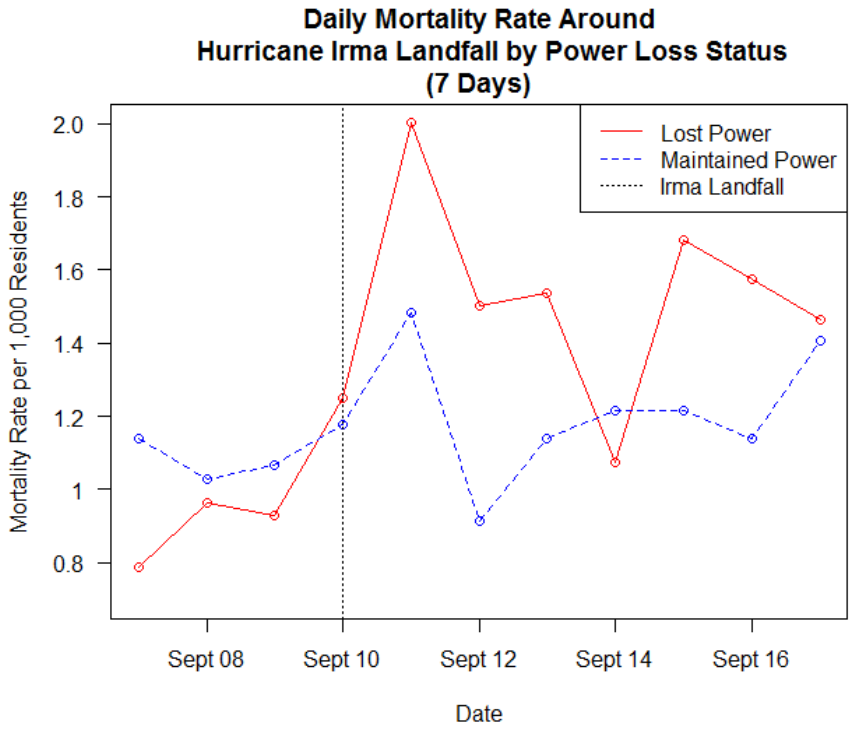


© 2021 Skarha J et al. *JAMA Health Forum.*

**eTable: Unadjusted and adjusted odds ratio from mixed effects model of first hospitalization within 30 days or mortality within 7 and 30 days among NH residents that experienced a power loss after Hurricane Irma compared to residents that did not experience a power loss[a,b]**

| | Unadjusted Odds Ratio 95% CI | Adjusted Odds Ratio 95% CI |
|---|---|---|
| First Hospitalization within 30 days | 1.03 (0.94, 1.12) | 1.05 (0.96, 1.14) |
| Mortality within 7 days | 1.24 (1.05, 1.47) | 1.24 (1.05, 1.47) |
| Mortality within 30 days | 1.09 (1.00, 1.21) | 1.12 (1.02, 1.23) |

[a]We used penalized quasilikelihood (PQL) Estimation of Generalized Linear Mixed Models with a random effect for nursing home ID.

[b]We adjusted for nursing home owner status (For-profit, Not-for-profit, Government-run), continuous bed count of nursing home beds, overall nursing home star rating (1-5), and rural county indicator.

© 2021 Skarha J et al. *JAMA Health Forum.*
